# Freshwater Salinization Syndrome Alters Nitrogen Transport in Urban Watersheds

**DOI:** 10.3390/w15223956

**Published:** 2023-11-09

**Authors:** Joseph G. Galella, Sujay S. Kaushal, Paul M. Mayer, Carly M. Maas, Ruth R. Shatkay, Shreeram Inamdar, Kenneth T. Belt

**Affiliations:** 1 Department of Geology & Earth System Science Interdisciplinary Center, University of Maryland, College Park, MD 20140, USA; 2 US Environmental Protection Agency Office of Research and Development, Center for Public Health and Environmental Assessment, Corvallis, OR 97333, USA; 3 Water Science and Policy Graduate Program, University of Delaware, Newark, DE 19716, USA; 4 Department of Geography and Environmental Systems, University of Maryland Baltimore County, 1000 Hilltop Circle, Baltimore, MD 21250, USA

**Keywords:** salinization, nitrogen, urban water quality

## Abstract

Anthropogenic salt inputs have impacted many streams in the U.S. for over a century. Urban stream salinity is often chronically elevated and punctuated by episodic salinization events, which can last hours to days after snowstorms and the application of road salt. Here, we investigated the impacts of freshwater salinization on total dissolved nitrogen (TDN) and ${{\mathrm{NO}}_{3}}^{-}/{{\mathrm{NO}}_{2}}^{-}$ concentrations and fluxes across time in urban watersheds in the Baltimore-Washington D.C. metropolitan area of the Chesapeake Bay region. Episodic salinization from road salt applications and snowmelt quickly mobilized TDN in streams likely through soil ion exchange, hydrologic flushing, and other biogeochemical processes. Previous experimental work from other studies has shown that salinization can mobilize nitrogen from sediments, but less work has investigated this phenomenon with high-frequency sensors and targeted monitoring during road salt events. We found that urban streams exhibited elevated concentrations and fluxes of TDN, ${{\mathrm{NO}}_{3}}^{-}/{{\mathrm{NO}}_{2}}^{-}$, and specific conductance that rapidly peaked during and after winter road salt events, and then rapidly declined afterwards. We observed plateaus in TDN concentrations in the ranges of the highest specific conductance values (between 1000 and 2000 μS/cm) caused by road salt events. Plateaus in TDN concentrations beyond a certain threshold of specific conductance values suggested source limitation of TDN in watersheds (at the highest ranges in chloride concentrations and ranges); salts were likely extracting nitrogen from soils and streams through ion exchange in soils and sediments, ion pairing in soils and waters, and sodium dispersion of soils to a certain threshold level. When watershed transport was compared across land use, including a forested reference watershed, there was a positive relationship between Cl^−^ loads and ${{\mathrm{NO}}_{3}}^{-}/{{\mathrm{NO}}_{2}}^{-}$ loads. This relationship occurred across all sites regardless of land use, which suggests that the mass transport of Cl^−^ and ${{\mathrm{NO}}_{3}}^{-}/{{\mathrm{NO}}_{2}}^{-}$ are likely influenced by similar factors such as soil ion exchange, ion pairing, sodium dispersion of soils, hydrologic flushing, and biogeochemical processes. Freshwater salinization has the potential to alter the magnitude and timing of total dissolved nitrogen delivery to receiving waters during winter months following road salt applications, and further work should investigate the seasonal relationships of N transport with salinization in urban watersheds.

## 1. Introduction

Freshwater salinization syndrome (FSS) has been observed throughout North America and Europe over the past century [1–3]. FSS refers to the suite of biophysical impacts triggered by increased salt ions in the environment. In temperate areas, anthropogenic salt inputs are thought to be the primary driver of FSS, with chronically elevated salinity punctuated by acute episodic salinization events caused by road salt application [4]. For example, U.S. sales of road salt have increased 147-fold from 149,000 metric tons in 1940 to nearly 22 million metric tons in the winter of 2017–2018 [5,6]. This rising trend may be due to impervious surface cover increasing across the United States largely due to increased suburbanization [7]. In addition, larger and more severe winter storms triggered by ongoing anthropogenic climate change may alter salinization trends [6]. FSS can cause alterations in contaminant mobility, biodiversity, and the quality of drinking water in urban watersheds [4,8–11]. Road salting mobilizes nutrients such as nitrogen (N) and phosphorus (P), organic matter, and other ions (Na^+^, Ca^2+^, Mg^2+^, K^+^, Cu, Mn, Sr^2+^) [2–4,9,12,13]. Less work has investigated the mobilization of N during and after road salt events. In this study, we use a combination of routine monitoring, winter storm event data, and high-frequency sensor data collected before, during, and after winter road salt events to understand temporal trends in N mobilization in urban watersheds.

Anthropogenically enhanced N loads can lead to eutrophication in urban waterways and coastal receiving waters [14–16]. N is often a limiting nutrient and can trigger the growth of phytoplankton and harmful algal blooms [14,17]. Consequently, much research has been devoted to understanding N transport from watersheds to streams [9,18,19]. Increases in N flux and concentration can increase during snowmelt events due to the flushing of N accumulated in soils over the dormant periods (from atmospheric deposition and/or soil nitrification) [18,20–22]. In addition, there can be natural seasonal pulses in N concentrations in streams that do not occur during winter. For example, N increases seasonally during summer in some streams in Maryland, and N is lowest and undetectable during the winter [23]; similar seasonal patterns with summer pulses are observed in some areas of North Carolina and Tennessee [24,25]. In urban watersheds, there can also be interactions between land use and hydrologic events to amplify N transport [15,26]. Recent work from laboratory experiments has shown that road salt applications in urban watersheds can mobilize N from soils [9,12]. Saltier soil conditions may also influence ammonium leaching in riparian groundwater at a site near a major roadway, but this was not monitored at the event scale or explicitly linked to road salt events [18].

The role of salinization impacts on watershed N transport and transformation has received relatively little attention (e.g., [9,12,27]). N concentrations in urban streams can be elevated by sewage leaks, atmospheric deposition, and fertilizers [28]. Our current understanding suggests that the ability of urban watersheds to retain and transform N may be diminished by: decreased hydrologic connectivity between streams and “hot spots” of N retention in urban riparian zones [29,30], increased hydrologic flashiness and decreased hydrologic residence times [31], headwater stream burial and channelization of streams [32–34], and decreased biological uptake of N following storms and scouring of biofilms [2]. Overall, there may be an increased supply of N in urban streams that exceeds biological demand [35]. Understanding the potential role of FSS in influencing and disrupting the N cycle is essential for accurately accounting for controls on N exports in urbanized watersheds [36–38].

Freshwater salinization enhances N transport from soils to streams via ion exchange, changes in the solubility of organic N, and changes in biogeochemical processes [12,13,38]. Ion exchange causes Na^+^ as well as other base cations (Ca^2+^, Mg^2+^, and K^+^) to desorb positively charged ${{\mathrm{NH}}_{4}}^{+}$ from sediments and negatively charged colloids [39–42]. Salt ions are very efficient at mobilizing ammonium (${{\mathrm{NH}}_{4}}^{+}$) and nitrate (${{\mathrm{NO}}_{3}}^{-}$) from soil samples [43,44]. Other salt ions can also mobilize elements via cation exchange (e.g., ammonium), anion exchange (e.g., nitrate), and solubility changes (e.g., dissolved organic N) [9,45]. NaCl, a common deicer, can also act to mobilize ammonium through cation exchange and sodium dispersion [46–49]. The positive charge of ammonium allows it to be sorbed onto negatively charged colloid particles in soils and sediments [50]. Influxes of base cations (Na^+^, Ca^2+^, and Mg^2+^) can then displace ammonium through base cation exchange, mobilizing ammonium into the water column [47,49]. Salt pollution can also influence the movement and extraction of other ions (such as nitrate) from soils and waters through ion pairing or temporary bonding by electrostatic forces. In addition, salt ions can influence the solubility of organic N [48], with some studies indicating that increased salinity can mobilize dissolved organic nitrogen (DON) from soil and plant matter [51]. There can also be “salting in” effects, which alter the organic matter’s quality and solubility [3,38]. During “salting in”, the solubility of organic matter is increased as ionic strength elevates [38].

Elevated salinity is detrimental to flora, both aquatic and terrestrial, causing injury through ion toxicity, osmotic stress, and nutrient deficiency via cation leaching by Na^+^ [40]. Salt-induced N mobilization may alter the balance of plant–microbe competition for nutrients, thus changing aquatic N removal rates [40]. Winter road salt pulses, which quickly increase stream water salinity, can also alter microbial processes, which affect the N cycle. High osmotic stress and ion-specific toxicity caused by elevated salinity can lead to cell lysis and the release of formerly stored inorganic and organic N and labile organic matter [38,40]. Hydrologic systems that have been chronically exposed to FSS may evolve microbial communities distinct from those in more natural watersheds [52]. These microbial communities are often adapted to elevated salinities [27,40,52].

TDN mobilization during the winter may also be enhanced by N buildup in soils and subsequent flushing during precipitation. This process, often observed in urban and forested catchments, occurs when upper soil layers are enriched with N during the low biological demand winter months [19,22,53,54] and flushed during periods of snowmelt or when the water table rises to saturate the N-laden horizon [54–56]. Markedly different patterns are observed in alternate climates or in areas of high agricultural land use [19,54].

Despite a variety of mobilization mechanisms in the environment, N concentrations and fluxes are often variable in their response to road salt applications in laboratory experiments [9,12]. In prior incubation studies, increased salinity showed a positive relationship with TDN concentration in some study sites, while other sites did not show a clear trend [9,12]. A possible explanation for this variability may be that the sediment present in some of the sites may have had a lower N content due to leaching from repeated previous exposure to elevated salinity levels [9,27]. In the environment, freshwater salinization may mobilize available N from watersheds and soils until there is source limitation or a plateau in concentrations at the highest levels of salinity [9,13]. Here, we investigate the potential for temporal patterns in the concentrations and mass fluxes of N to be exacerbated by FSS at the watershed scale. We hypothesize that episodic winter salt pollution has the potential to mobilize N from urban watersheds and further increase N concentrations and fluxes.

## 2. Methods

### 2.1. Study Sites

**Figure 1.**
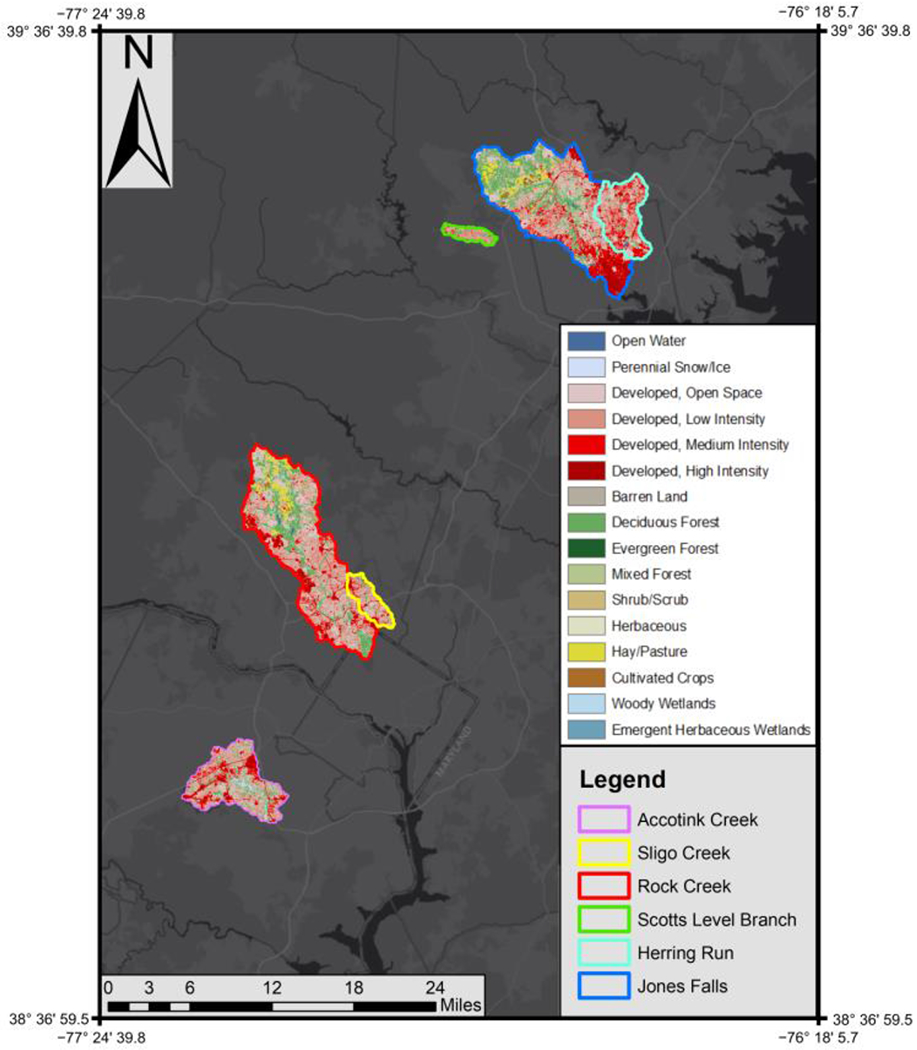
Land use at Herring Run, Jones Falls, Scotts Level Branch, Rock Creek, Sligo Creek, and Gwynns Falls watersheds in the Baltimore, MD and Washington DC metro areas, United States of America.

Our study sites consisted of five urban streams within the Chesapeake Bay watershed. Rock Creek and Sligo Creek are found within the D.C. metropolitan area, while Scotts Level Branch, Herring Run, and Jones Falls are contained within the Baltimore metropolitan area (Table 1; Figure 1). We chose these sites because of access to high-frequency continuous sensor data, which measured specific conductance and discharge from USGS stations. Sites were also proximal to the University of Maryland College Park, where further analysis was conducted. Urban and suburban land use is most prevalent at all sampling sites, with impervious surface cover varying between 18–33% along the five streams. Riparian buffers are present at all sites but are most pronounced (wider and more contiguous) at Rock Creek and least pronounced at Jones Falls, where they become absent within the city of Baltimore where the channel is buried. Jones Falls is also notable as the only tidally influenced site. StreamStats, a USGS-based web application, was used to delineate watersheds, while the National Land Cover Database provided 30 m resolution land use and impervious surface cover data [57,58]. Watershed characteristics were visualized with ArcMap 10.8.2, ESRI 2022 ArcGIS Desktop, Redlands, CA, USA.

### 2.2. Field Measurements

#### Routine Monitoring and High-Frequency Monitoring

Routine stream water grab samples were collected at bi-weekly intervals (every other week) beginning in October 2017 and ending between 2019 and 2021 (Table 1). Samples were collected directly into 125 mL HDPE Nalgene acid-washed sample bottles [4]. For targeted winter storm event sampling, hourly samples were collected using an automated sampler (ISCO 3700) at Sligo Creek [59]. The automated sampler was used to collect samples before, during, and slightly after snowstorm events. Every hour, 300 mL of water was collected and remained on ice (internal reservoir) until the storm subsided and it was safe to retrieve the unit(s) [4,13]. Once collected, samples were kept chilled on ice in the field or in a lab fridge kept at 4 ± 2 °C until analysis within 14 days [4,13]. If samples were not analyzed within 14 days, they were frozen to preserve them for future analysis.

All sampling sites were located at USGS gauge stations equipped with high-frequency sensor measurements of discharge and SC (5–15 min) [60]. USGS monitoring stations utilize multiparameter datasondes, which take instantaneous measurements. Rock Creek (USGS Gauge Station 1648010), Sligo Creek (USGS Gauge Station 1650800), Scotts Level Branch (USGS Gauge Station 1589290), Herring Run (USGS Gauge Station 1585219), and Jones Falls (USGS Gauge Station 1589485) were all monitored continuously for discharge (Q) and specific conductance (SC). Rock Creek was also continuously monitored by sensors for ${{\mathrm{NO}}_{3}}^{-}/{{\mathrm{NO}}_{2}}^{-}$ [60] through February 2019, after which the ${{\mathrm{NO}}_{3}}^{-}/{{\mathrm{NO}}_{2}}^{-}$ sensor was discontinued. Continuous SC, Q, and ${{\mathrm{NO}}_{3}}^{-}/{{\mathrm{NO}}_{2}}^{-}$ data from Accotink Creek near Annandale, VA (USGS Gauge Station 1654000), 23 km from Rock Creek, was used to supplement the ${{\mathrm{NO}}_{3}}^{-}/{{\mathrm{NO}}_{2}}^{-}$ dataset after measurements ceased at Rock Creek [60]. SC was reported in microsiemens per centimeter (μS/cm) at 25 °C. SC contact sensors were temperature adjusted (to report at 25 °C) and accurate > 0.5 ± 0.5% of readings, or ±2 μS/cm [61,62]. Temperature data was collected using a thermistor with an accuracy of ±0.1 °C. Q was measured to an accuracy of the nearest 0.01 ft or 0.2% stage height [62,63]. Instantaneous measurements of ${{\mathrm{NO}}_{3}}^{-}/{{\mathrm{NO}}_{2}}^{-}$ were collected via a multiparameter datasonde [60]. Information regarding the instrumental error, calibrations, and accuracy of USGS multiparameter datasondes are further described in [61,62].

### 2.3. Laboratory

#### 2.3.1. Water Quality Analyses

Once transported to the laboratory, water samples were immediately filtered through an ashed Whatman 0.7-micron glass fiber filter into labeled acid-washed HDPE Nalgene bottles [4,13]. Total dissolved nitrogen (TDN) was analyzed on a Shimadzu total organic carbon analyzer (Shimadzu TOC-L CPH/CPN) total nitrogen module, TNM-1 using a chemiluminescence method [4,13,64]. Calibration of the TOC-L was performed every run (max 80 samples) with a 5-point calibration curve. Blanks were run and checked for accuracy (<0.5) every 15 samples [4,13]. Calibration (internal) standards as well as external check standards were used in instrument calibration to determine if results were within the acceptable range of ±20% of the true value [4,13].

#### 2.3.2. Numerical Analysis and Plotting

MATLAB 2021b was used to generate hysteresis plots. Hysteresis plots are used to assess how the concentration and flux of nutrients evolve over a precipitation event. SC in microsiemens per centimeter at 25 °C was plotted on the *x* axis. High-frequency storm data collected at Sligo Creek plotted TDN on the Y axis in milligrams per liter. Data collected at Accotink Creek at the USGS monitoring site plotted ${{\mathrm{NO}}_{3}}^{-}/{{\mathrm{NO}}_{2}}^{-}$ on the Y axis. Data from Rock Creek was not used in this analysis due to ${{\mathrm{NO}}_{3}}^{-}/{{\mathrm{NO}}_{2}}^{-}$ sensor cessation in 2019. Four winter precipitation events from 2019 to 2021 were used for hysteresis analysis and a color scale was added to the data points in order to show the temporal nature of the data. Blue data points were taken near the beginning of the precipitation event and yellow data points were collected at the end. Hysteresis loops moving in a clockwise direction indicated that N or ${{\mathrm{NO}}_{3}}^{-}/{{\mathrm{NO}}_{2}}^{-}$ concentrations decreased with increasing SC over the course of the storm. Counterclockwise hysteresis indicated that N or ${{\mathrm{NO}}_{3}}^{-}/{{\mathrm{NO}}_{2}}^{-}$ concentrations increased with increasing SC over the course of the storm.

TDN and ${{\mathrm{NO}}_{3}}^{-}/{{\mathrm{NO}}_{2}}^{-}$ mass fluxes in kg/h were calculated using the following formula:

$$\left(\text{TDN or}\;{{\mathrm{NO}}_{3}}^{-}/{{\mathrm{NO}}_{2}}^{-}\;\text{concentration in mg}/\text{L}\right)\times \left(\text{Q in CFS}\right)\times 28.3168\times 60\times 60/1,000,000$$

where 28.3168 is liters per cubic foot, 60 is seconds per minute, 60 is minutes per hour, and 1,000,000 is milligrams per kilogram.

## 3. Results

### 3.1. Seasonal Trends in Routine Sampling Sites and USGS Data

**Figure 2.**
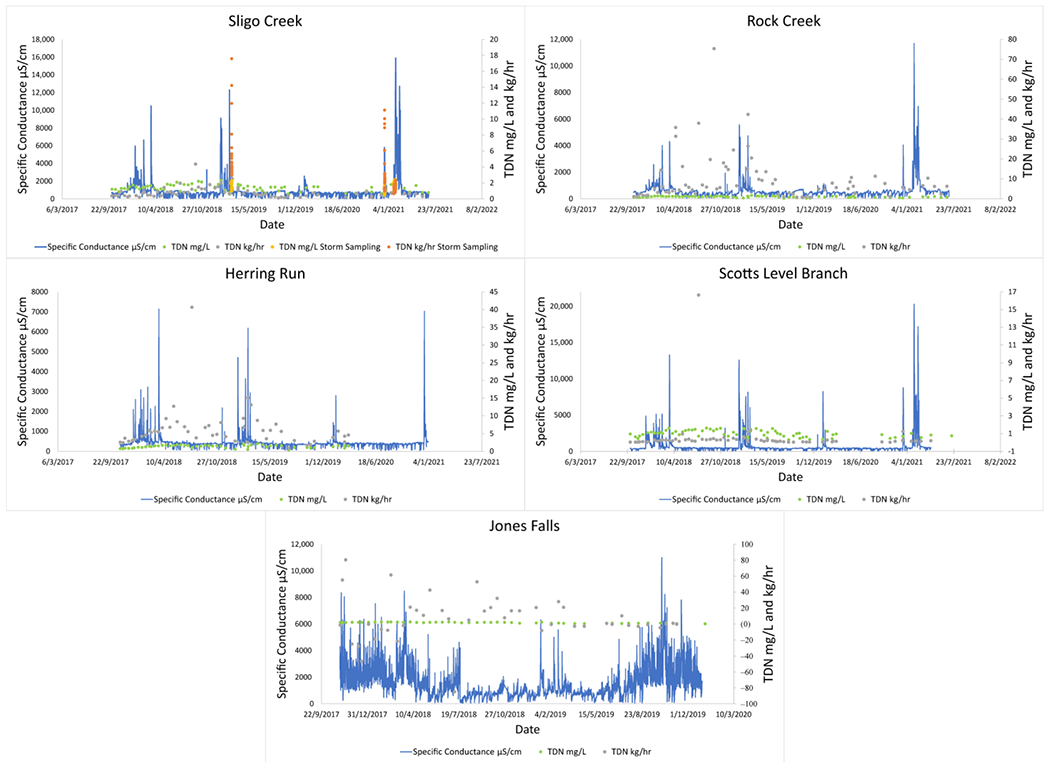
Elevated TDN peaks were associated with peaks in specific conductance. Rock Creek, Sligo Creek, Herring Run, Scotts Level Branch, and Jones Falls were monitored by bi-weekly sampling. Targeted storm sampling data were also included at Sligo Creek. Concentration (mg/L) and flux kg/h were plotted. Negative values noted at Jones Falls are when flow is reversed due to tidal influence.

In situ sensor and bi-weekly grab sampling data revealed the existence of distinct seasonal water quality patterns in streams draining all five watersheds in the Baltimore and Washington D.C. metro areas. Sensor data from the USGS showed that winter road salting could regularly increase SC by more than an order of magnitude over the course of several hours (Table 2, Figure 2). Peaks in TDN concentrations and fluxes were also elevated during winter months, coinciding with the peaks in SC (Figure 2). All non-tidal routine sampling sites (Sligo Creek, Rock Creek, Herring Run, and Scotts Level Branch) had coinciding peaks in specific conductance and TDN concentrations or fluxes over time (Figure 2). Jones Falls did not have coinciding peaks in SC and TDN concentrations; this is probably because the site is tidal, which results in highly variable SC readings throughout the year. There were elevated TDN mass fluxes at Rock Creek during 2018, which are likely a result of sewage leaks frequently reported in the area, requiring a fecal bacteria TMDL to be created for the catchment [65].

**Figure 3.**
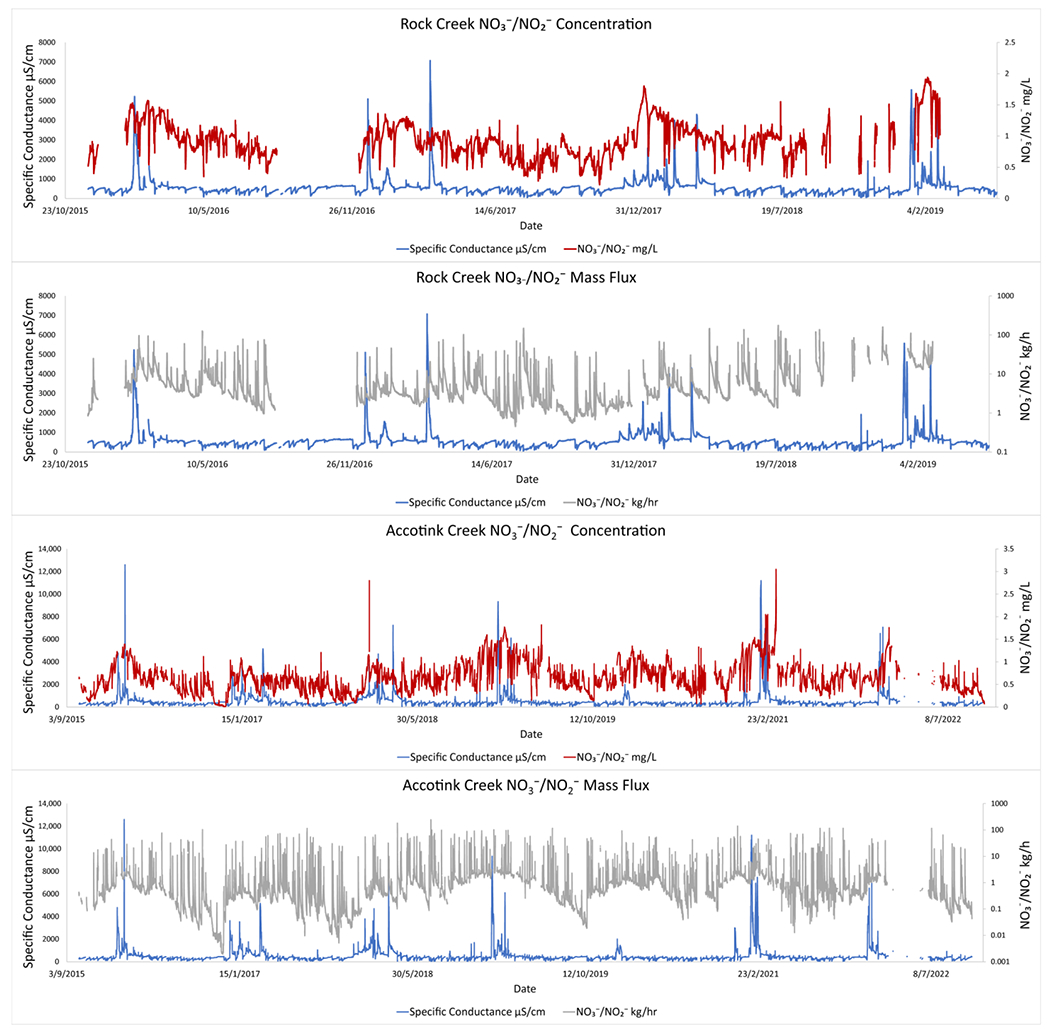
Peaks in ${{\mathrm{NO}}_{3}}^{-}/{{\mathrm{NO}}_{2}}^{-}$ concentrations associated with peaks in specific conductance. Concentrations of ${{\mathrm{NO}}_{3}}^{-}/{{\mathrm{NO}}_{2}}^{-}$ (mg/L) were measured by high-frequency sensors at Rock Creek and Accotink Creek and hourly fluxes (kg/h) were calculated from discharge and concentration data.

USGS data from Rock Creek and Accotink Creek also showed similar coinciding pulses in SC and ${{\mathrm{NO}}_{3}}^{-}/{{\mathrm{NO}}_{2}}^{-}$ (Figure 3). Seasonal trends were most apparent when comparing mass fluxes of ${{\mathrm{NO}}_{3}}^{-}/{{\mathrm{NO}}_{2}}^{-}$ with SC at Accotink Creek (Figure 3). Peak SC values were always associated with peak mass fluxes of ${{\mathrm{NO}}_{3}}^{-}/{{\mathrm{NO}}_{2}}^{-}$ every winter from 2015 to 2021. Elevated hourly mass fluxes of TDN and ${{\mathrm{NO}}_{3}}^{-}/{{\mathrm{NO}}_{2}}^{-}$ coincided with peaks in SC, which suggested that N concentrations were not significantly diluted by increasing discharge (Figures 2 and 3).

### 3.2. Targeted Storm Event Sampling of Winter Precipitation (Sligo Creek)

**Figure 4.**
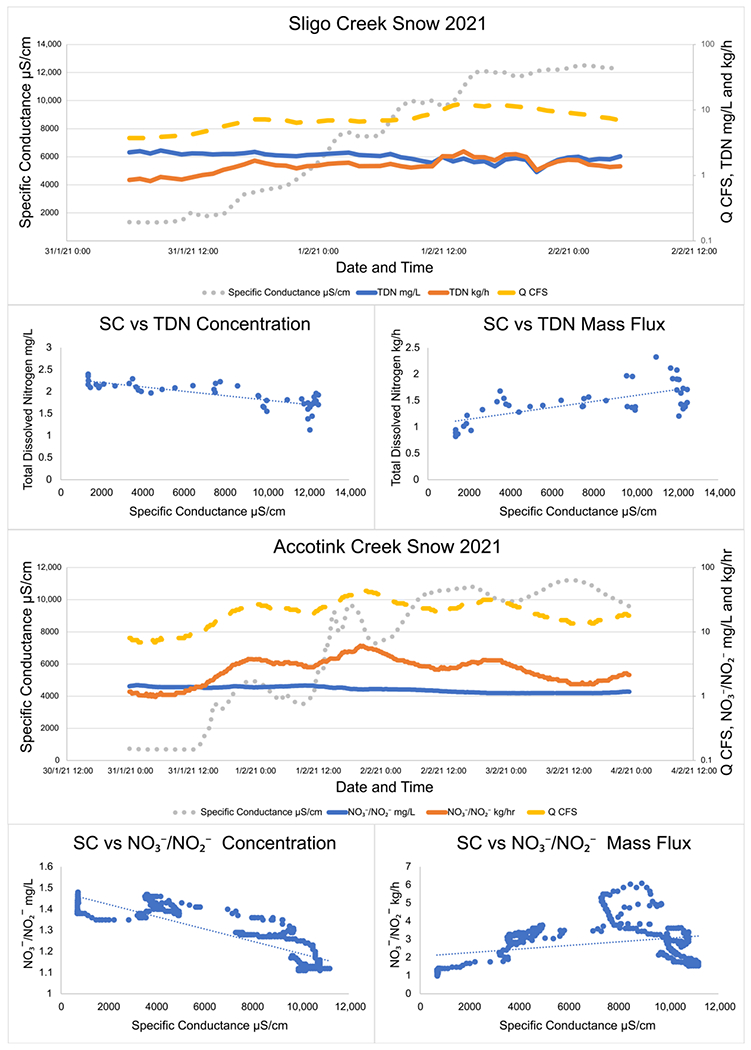
Specific conductance, TDN, ${{\mathrm{NO}}_{3}}^{-}/{{\mathrm{NO}}_{2}}^{-}$, and discharge trends during two snowstorms at Sligo Creek and Accotink Creek. Over the course of the storm, concentrations of TDN and ${{\mathrm{NO}}_{3}}^{-}/{{\mathrm{NO}}_{2}}^{-}$ were found to decrease while mass flux was found to increase. TDN data were collected by targeted storm sampling and ${{\mathrm{NO}}_{3}}^{-}/{{\mathrm{NO}}_{2}}^{-}$ data were collected by high-frequency sensor measurements. Linear regression lines are plotted through all ${{\mathrm{NO}}_{3}}^{-}/{{\mathrm{NO}}_{2}}^{-}$ vs. specific conductance plots to make the visualization of trends easier.

Analysis of high-frequency sampling conducted during winter precipitation events illustrated two different patterns in watershed TDN and ${{\mathrm{NO}}_{3}}^{-}/{{\mathrm{NO}}_{2}}^{-}$ transport. When a winter storm was dominated by solid precipitation (snow/sleet) and road salt concentrations consistently increased, concentrations of TDN and ${{\mathrm{NO}}_{3}}^{-}/{{\mathrm{NO}}_{2}}^{-}$ decreased with increasing SC. For example, during a snowstorm starting on 31 January 2021 at Sligo Creek, TDN concentration dropped from 2.35 mg/L at the start of the storm to 1.128 mg/L at the end (Figure 4). However, hourly mass fluxes consistently increased with increased SC throughout the snowstorm event from 0.82 kg/h TDN when the storm started to 2.33 kg/h TDN as the winter storm ceased. Peak SC reached as high as 12,400 μS/cm by the end of the snowstorm event. These observations are consistent across multiple different snowstorms and at both Sligo Creek and Accotink Creek (Figure 4).

**Figure 5.**
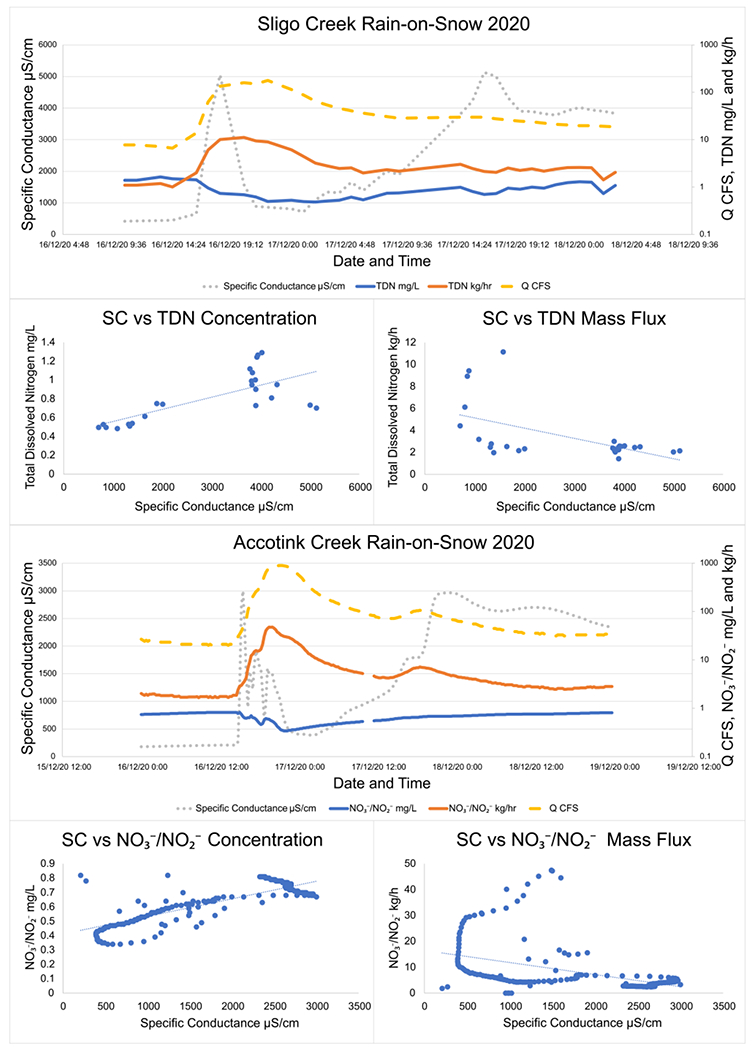
Specific conductance, TDN, ${{\mathrm{NO}}_{3}}^{-}/{{\mathrm{NO}}_{2}}^{-}$, and discharge trends during two rain-on-snow storms at Sligo Creek and Accotink Creek. Over the course of the storm, concentrations of TDN and ${{\mathrm{NO}}_{3}}^{-}/{{\mathrm{NO}}_{2}}^{-}$ were found to increase while mass fluxes were found to decrease. TDN data were collected by targeted storm sampling and ${{\mathrm{NO}}_{3}}^{-}/{{\mathrm{NO}}_{2}}^{-}$ data were collected by high-frequency sensor measurements. Linear regression lines are plotted through all ${{\mathrm{NO}}_{3}}^{-}/{{\mathrm{NO}}_{2}}^{-}$ vs. specific conductance plots to make the visualization of trends easier.

Interestingly, winter storms which began as snow and then shifted to rain (rain-on-snow events), have different patterns. During a rain-on-snow event captured with targeted storm sampling at Sligo Creek (Figure 5), SC quickly peaked (within 3 h) at 5000 μS/cm and then sharply decreased, as rain diluted the discharge to 890 μS/cm within three hours. As the rain transitioned back to snow, the SC rose again to 5000 μS/cm. During this rain-on-snow event, TDN concentrations rose with increased SC from 0.49 mg/L to 1.29 mg/L, but hourly TDN fluxes decreased from 11.1 kg/h to 2.1 as SC rose.

### 3.3. Hysteresis of Nitrogen Concentrations during Winter Snow Events

**Figure 6.**
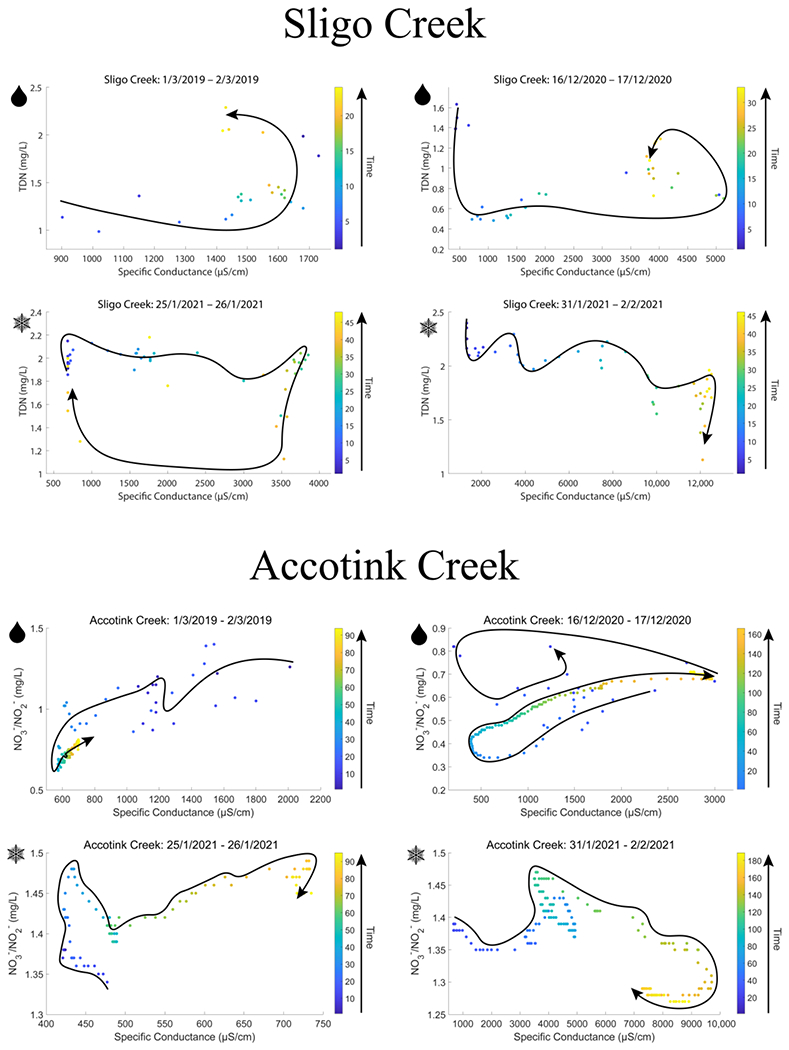
Hysteresis loops for snow and rain-on-snow events at Sligo Creek and Accotink Creek. Sligo Creek data recorded TDN concentrations from targeted storm sampling measurements and Accotink data recorded ${{\mathrm{NO}}_{3}}^{-}/{{\mathrm{NO}}_{2}}^{-}$ concentrations from high-frequency sensor measurements. Corresponding high-frequency sensor data for ${{\mathrm{NO}}_{3}}^{-}/{{\mathrm{NO}}_{2}}^{-}$ from Rock Creek during the same time period were not available due to ${{\mathrm{NO}}_{3}}^{-}/{{\mathrm{NO}}_{2}}^{-}$ sensor cessation at that location in 2019. Clockwise loops occurred during snow events and counterclockwise loops occurred during rain-on-snow events at both sites. Snowflake symbols denote a snow-only precipitation event, raindrop symbols denote a rain-on-snow event.

Hysteresis analyses of rain-on-snow vs. snow events suggested distinct patterns of TDN mobilization. Figure 6 shows hysteresis loops from two rain-on-snow events (indicated with the raindrop symbol) and two snow events (indicated with the snowflake symbol). Counterclockwise hysteresis was observed for both rain-on-snow events at Sligo Creek (2019 and 2020), which indicated that TDN concentrations increased with increasing SC values over time. Clockwise hysteresis was observed for both snow events recorded at Sligo Creek (2021), which indicated that TDN concentrations decreased with increasing SC values over time. As expected, the results were consistent with those at nearby Accotink Creek, where USGS sensors also measured high-frequency ${{\mathrm{NO}}_{3}}^{-}/{{\mathrm{NO}}_{2}}^{-}$ concentrations. Counterclockwise hysteresis was observed during both rain-on-snow events and clockwise hysteresis was observed during both snowstorms. The 2020 rain-on-snow event at Accotink Creek was unique, as it had both a clockwise and counterclockwise loop present.

### 3.4. Plateaus in N Concentrations over Time during Road Salt Events

**Figure 7.**
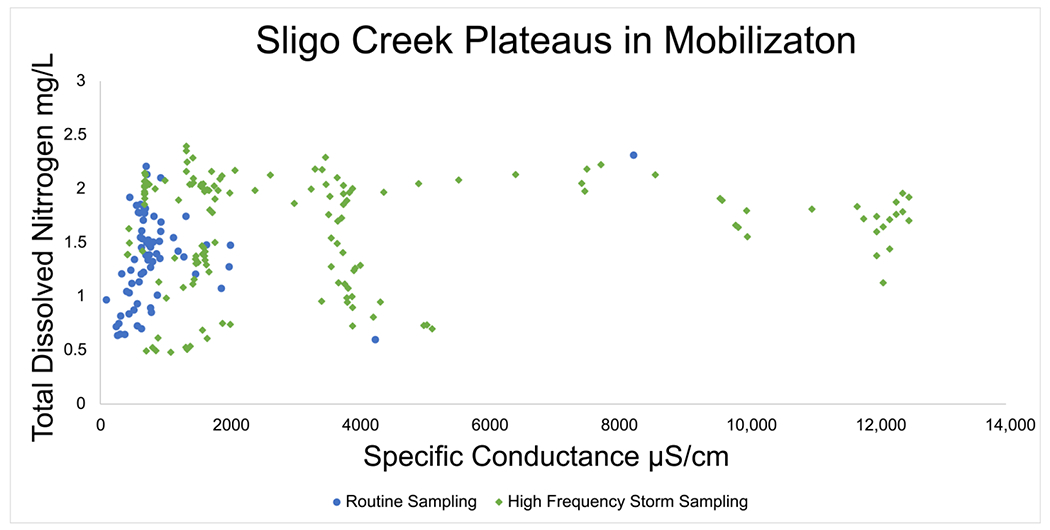
Specific conductance at Sligo Creek. Regular bi-weekly sampling is plotted in blue circles and targeted winter storm event sampling is plotted in green diamonds. A plateau in mobilization is notable at ~2000 μS/cm where concentrations of TDN stop increasing with increases in specific conductance.

**Figure 8.**
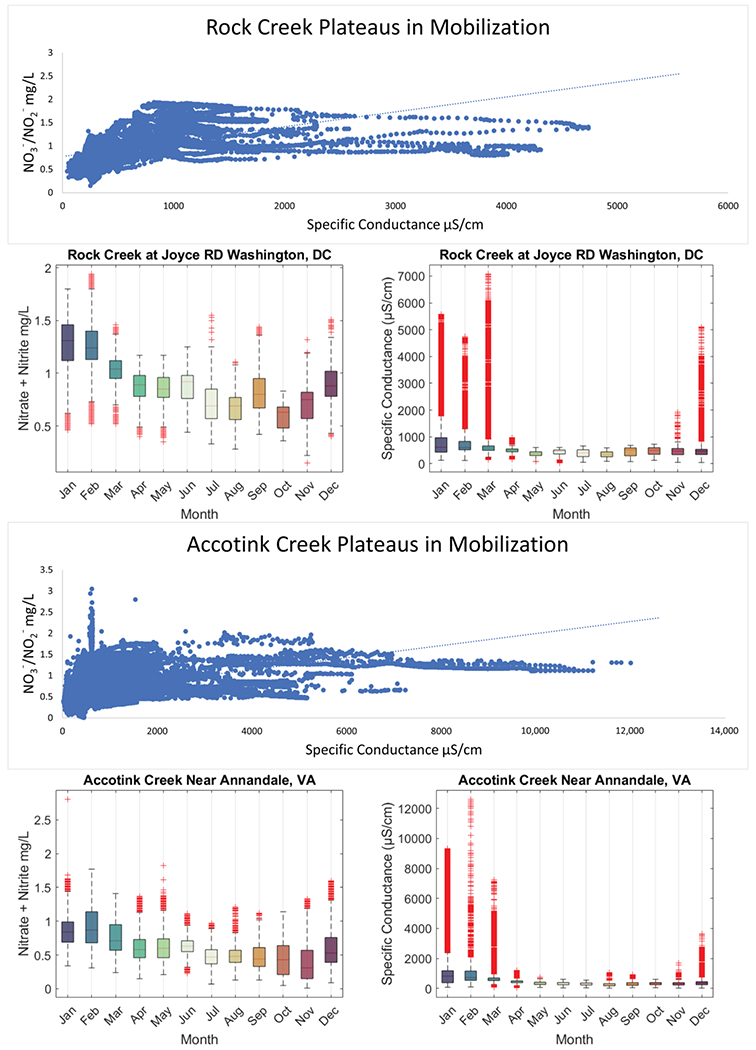
Specific conductance is plotted against ${{\mathrm{NO}}_{3}}^{-}/{{\mathrm{NO}}_{2}}^{-}$ concentration (mg/L) for Rock Creek and Accotink Creek from high-frequency sensor measurements. Data from Rock Creek and Accotink Creek both show distinctive plateaus in ${{\mathrm{NO}}_{3}}^{-}/{{\mathrm{NO}}_{2}}^{-}$ mobilization at ~1000 μS/cm. Boxplots show monthly data for both sites where both ${{\mathrm{NO}}_{3}}^{-}/{{\mathrm{NO}}_{2}}^{-}$ and specific conductance peak during the winter and reach a minimum in the autumn.

Data from routine and targeted snow event monitoring at Sligo Creek (Figure 7) showed a positive relationship between SC and TDN. The increasing trend between SC and TDN was present until ~2000 μS/cm, where a plateau was reached. Pearson correlation was 0.242 for routine monitoring data (*p* 0.04) and 0.08 for high-frequency data (*p* 0.33), illustrating the plateau effect above ~2000 μS/cm. A similar pattern was also observed using USGS data from Rock Creek and Accotink Creek, where a positive correlation between SC and ${{\mathrm{NO}}_{3}}^{-}/{{\mathrm{NO}}_{2}}^{-}$ concentrations was observed until a plateau at ~1000 μS/cm (Figure 8). Pearson correlation was 0.643 at Rock Creek (*p* < 0.0001) for specific conductance <1000 μS/cm and −0.38 (*p* < 0.0001) for specific conductance > 1000 μS/cm. Pearson correlation was 0.32 at Accotink Creek (*p* < 0.0001) for specific conductance < 1000 μS/cm and 0.131 (*p* < 0.0001) for specific conductance > 1000 μS/cm. Seasonal trends are shown for Rock Creek and Accotink Creek via box-and-whisker plots. Points above the upper quartile marked in red show elevated SC values present during the winter months of December through March. Elevated ${{\mathrm{NO}}_{3}}^{-}/{{\mathrm{NO}}_{2}}^{-}$ is also present during the same timeframe of December through March at both Rock Creek and Accotink Creek (Figure 8).

## 4. Discussion

Our results suggest that FSS can alter the concentrations and fluxes of N transported from urban watersheds. Many studies have evaluated other causes of elevated N transport from watersheds related to atmospheric deposition, land use, management, and other factors [14,23,30,40]. Our work also suggests that relationships between TDN or ${{\mathrm{NO}}_{3}}^{-}/{{\mathrm{NO}}_{2}}^{-}$ concentrations and specific conductance can reveal unique insights into N cycling and transport in urban environments. When targeted winter storm event data and high-frequency sensor data during winter storms are analyzed (along with routine monitoring data throughout the year), a distinct pattern emerges; there are plateaus in N concentrations at relatively high specific conductance. Up to a certain threshold, the effects of FSS in certain urban catchments can act to elevate both TDN and ${{\mathrm{NO}}_{3}}^{-}/{{\mathrm{NO}}_{2}}^{-}$ concentrations, as well as hourly mass fluxes. This is observable as seasonal pulses (Figures 2, 3 and 8) during and after winter storm events (Figures 4 and 5). Complex biogeochemical interactions can drive the mobilization, transport, and retention of N. However, there were clear patterns in winter pulses of N concentrations and fluxes in this study, which suggest the importance of water quality monitoring in response to road salt events during winter months should be reconsidered.

### 4.1. FSS and Mobilization of TDN and ${{\mathrm{NO}}_{3}}^{-}/{{\mathrm{NO}}_{2}}^{-}$ Pulses

At routine sampling sites, TDN and ${{\mathrm{NO}}_{3}}^{-}/{{\mathrm{NO}}_{2}}^{-}$ concentrations and hourly mass fluxes increased throughout the winter, especially coinciding with winter precipitation events and associated peaks in SC (Figures 2 and 3). Statistically significant linear relationships between SC and TDN were most apparent at Sligo Creek, where targeted winter storm event sampling was conducted. Similar trends were observed at Rock Creek and Accotink Creek where high-frequency data was also available. This highlights the importance of integrating storm event or high-frequency temporal sampling in monitoring programs ([4]), although this data is often difficult to collect. Average hourly mass fluxes of 0.8 kg/h TDN were observed at Sligo Creek during routine sampling and peaked at 2.5 kg/h. Targeted winter storm event sampling at Sligo Creek during the 2019 snow event captured hourly TDN mass fluxes reaching a maximum of 17.6 kg/h (within a one hour of peak SC (2050 μS/cm). Positive SC and TDN relationships were also observed at Rock Creek, Scotts Level Branch, and Herring Run. Data from USGS continuous monitoring sensors at Rock Creek and Accotink Creek showed seasonal patterns in ${{\mathrm{NO}}_{3}}^{-}/{{\mathrm{NO}}_{2}}^{-}$ mobilization, with peaks in ${{\mathrm{NO}}_{3}}^{-}/{{\mathrm{NO}}_{2}}^{-}$ concentrations and hourly mass fluxes, which coincided with peaks in specific conductance (Figures 2 and 3).

Many previous studies have characterized seasonal patterns in N concentrations in streams. For example, studies from different regions throughout the U.S. have shown that ${{\mathrm{NO}}_{3}}^{-}$ and TDN peak during winter months, when biological activity is lowest [66,67]. However, there are exceptions where stream N concentrations can peak during summer months in some watersheds in warmer regions due to increasing organic matter decomposition, N mineralization, and nitrification [25,68]. In the Chesapeake Bay watershed, Testa et al. 2018, proposes that there are seasonal cycles to N loads throughout the year. During spring and early summer, there are high ${{\mathrm{NO}}_{3}}^{-}$, ${{\mathrm{NH}}_{4}}^{+}$, and particulate N inputs, leading to a bloom of phytoplankton during the spring and oxygen depletion and ${{\mathrm{NH}}_{4}}^{+}$ accumulation in the summer [16]. During late summer and autumn, phytoplankton production is reduced and vertical mixing of water columns resumes, replenishing dissolved oxygen and increasing nitrification. This process may explain some of the seasonal cycling observed in this study (Figures 2,3 and 8) but peaks associated with road salting events and positive correlations between specific conductance and ${{\mathrm{NO}}_{3}}^{-}/{{\mathrm{NO}}_{2}}^{-}$ mobilization show that FSS can play a role in N cycling and transport.

Our results are consistent with previous laboratory studies that show that salinization can alter N concentrations in soils, streams, and wetlands [9,12,27,48]. Lab-based incubation analysis on samples collected from the D.C. and Baltimore metro areas showed increasing mobilization of TDN with increasing salinity at 75% of the sites sampled [9]. Suburban and urban sites located in the Gwynns Falls watershed showed positive linear relationships with increasing salinization (Figure S1) [9].

Impervious surface cover and urban land use have also been linked to total Kjeldahl nitrogen (TKN) mobilization under FSS conditions. TKN increased in concentration as salinity increased at all sites studied (forested, agricultural, suburban, and urban), with the greatest degree of mobilization recorded at urban sites [12]. The same trend occurred when mobilization was graphed over watershed percent impervious surface cover (% ISC) instead of using land use classifications. Significant positive relationships were discovered between % ISC and TKN concentration [12].

Mobilization of N and ${{\mathrm{NO}}_{3}}^{-}/{{\mathrm{NO}}_{2}}^{-}$ is thought to be driven chemically by a combination of ion exchange [43,44], ion pairing, and sodium dispersion [46–49]. Cation exchange has also been shown to mobilize ${{\mathrm{NH}}_{4}}^{+}$ from soils and sediments into solution [40]. Biological factors like cell lysis due to rapidly changing osmotic stress and ion-specific toxicity may also contribute to additional N loading during acute road salting events [38,40]. The rates of microbial processes which control N transformation may also be affected by elevated salinities associated with FSS, increasing leaching of ${{\mathrm{NO}}_{3}}^{-}$ from impacted waterways [48]. However, some microbial communities in watersheds may become adapted to elevated salinities, lessening the cell lysis associated with high osmotic stress [27]. More work is necessary to investigate the effects of increased salinization on microbial N cycling.

### 4.2. Concurrent N Build and Flush in Temperate Watersheds

Some degree of the increased TDN and ${{\mathrm{NO}}_{3}}^{-}/{{\mathrm{NO}}_{2}}^{-}$ fluxes recorded during winter snow events may be caused by the buildup and flushing of N in the upper layers of soil present at the research sites. Urban and forested watersheds in temperate climates are known to accumulate N during the winter months when microbes and flora are least active [19,22,53,54]. However, the results suggest that this seasonal mobilization with precipitation is enhanced by mobilization via interactions with deicers. Across varied land use at Urban (Rock Creek), Rural (Connecticut River), and Agricultural (Maumee River) sites, there were significant (*p* < 0.05) positive relationships between chloride (Cl^−^) mass flux and ${{\mathrm{NO}}_{3}}^{-}$ mass flux, indicating that build and flush is not the only mechanism driving nutrient mobilization.

### 4.3. High Temporal Frequency Sampling and Storm Hysteresis

Different types of winter precipitation can affect TDN and ${{\mathrm{NO}}_{3}}^{-}/{{\mathrm{NO}}_{2}}^{-}$ mobility in urban catchments. High-frequency sampling at Sligo Creek and Accotink Creek showed that during solid precipitation events, TDN and ${{\mathrm{NO}}_{3}}^{-}/{{\mathrm{NO}}_{2}}^{-}$ concentrations decreased (clockwise hysteresis) while mass flux increased over time (Figures 3 and 4). The opposite trends were found during rain-on-snow events, where N and ${{\mathrm{NO}}_{3}}^{-}/{{\mathrm{NO}}_{2}}^{-}$ concentrations increased (counterclockwise hysteresis) while mass flux decreased over time.

During rain-on-snow events, at the point of transition from snow to rain, hourly mass fluxes of TDN and ${{\mathrm{NO}}_{3}}^{-}/{{\mathrm{NO}}_{2}}^{-}$ were significantly higher than at any other point during the storm. These peaks were captured by both high-frequency monitoring by sensors and targeted storm sampling. Initial N mobilization by elevated SC, followed by elevated discharge, caused mass fluxes to briefly reach 47 kg/h ${{\mathrm{NO}}_{3}}^{-}/{{\mathrm{NO}}_{2}}^{-}$ at Accotink Creek (Figure 5). Similar conditions have been recorded in rural forested catchments in Ontario, Canada. During individual rain-on-snow events, as much as 40% of the annual ${{\mathrm{NO}}_{3}}^{-}$ export from the catchment could be mobilized [69]. These significant ${{\mathrm{NO}}_{3}}^{-}$ pulses were often followed by depressed pH and alkalinity [69].

After the transition from snow to rain, decreasing specific conductance associated with rainfall may limit the mobilization of TDN into the water column via hydrologic dilution, diminished base cation exchange, or decreased osmotic stress on microbial cell membranes [9,40,70]. We speculate that increased N mass flux with increased SC during snow events may be a result of freshwater and saltwater mixing in the hyporheic zone and the mobilization of N from transient storage zones. When a high density gradient is present (saline water mixing with freshwater), the mixing efficiency and reaction area increase, which can elevate ${{\mathrm{NO}}_{3}}^{-}$ production from the lysis of microbial cells, ammonification and nitrification, and subsequent transport. [71]. However, further research is necessary to determine why N fluxes and concentrations increase due to hydrological, physical, and biological mechanisms.

### 4.4. Plateaus in N Concentrations over Time during Road Salt Events

When plotted against SC, TDN and ${{\mathrm{NO}}_{3}}^{-}/{{\mathrm{NO}}_{2}}^{-}$ concentrations have a positive relationship until they reach a plateau. This plateau was found to be at ~1000 μS/cm for ${{\mathrm{NO}}_{3}}^{-}/{{\mathrm{NO}}_{2}}^{-}$ concentrations (Figure 8) and closer to 2000 μS/cm for N concentrations (Figure 7). Thresholds in N species mobilization by FSS are thought to be caused by the cation exchange capacity (CEC) of the sediments present at these catchments (Sligo Creek, Rock Creek, and Accotink Creek). Similar CEC limits were found when examining major and trace element mobilization at urban stormwater best management practices [13]. Plateaus in N mobilization were also discovered in salinization incubation experiments in Haq et al., 2018. Once a cation like Na^+^ has mobilized positively charged species like ${{\mathrm{NH}}_{4}}^{+}$ from all exchange sites available on sediment colloids, the concentration can no longer increase [47,49]. Thresholds in N and ${{\mathrm{NO}}_{3}}^{-}/{{\mathrm{NO}}_{2}}^{-}$ mobilization can also be influenced by biological mechanisms. Recent studies have suggested that over decades of exposure to FSS, microbial communities may become adapted to the high osmotic stress of episodic FSS due to road salting, leading to reduced cell lysis andNdispersion at high salinities [27,40]. These biological factors may also play a role in determining the limits of the plateaus observed.

### 4.5. Nitrate and Chloride Fluxes across Land Use

**Figure 9.**
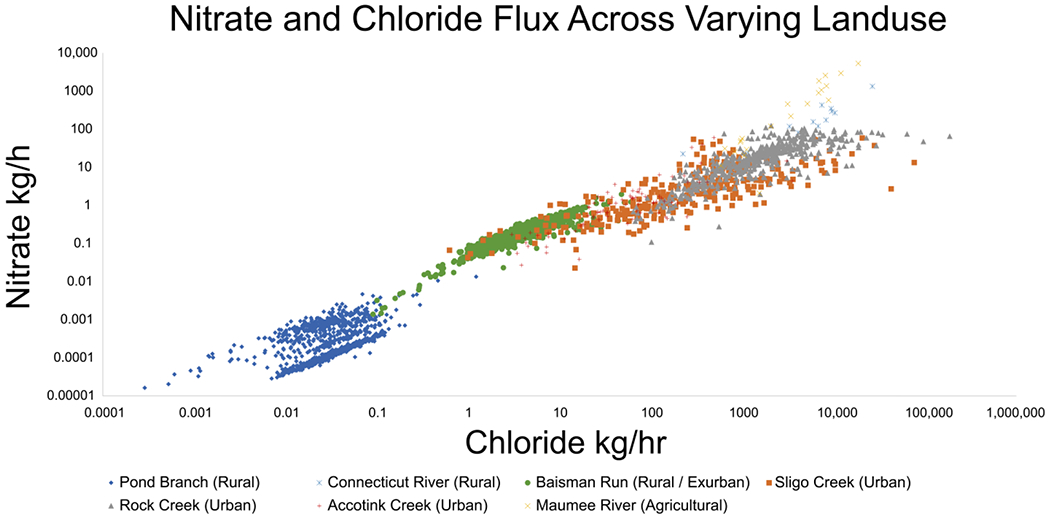
${{\mathrm{NO}}_{3}}^{-}$ and Cl^−^ fluxes show co-mobilization. Flux data from Connecticut River, Sligo Creek, Rock Creek, Accotink Creek, and Maumee River are estimated from concentration and streamflow data from USGS gauge numbers 01161280, 01650800, 01648010, 01654000, and 04183500, respectively. Flux data from Pond Branch and Baisman Run are estimated from concentration data from the Baltimore Ecosystem Study [72] and streamflow data from USGS gauge numbers 01583570 and 01583580, respectively. Fluxes are lowest at rural sites, increasing through urban sites, and reaching a peak at agricultural sites. Anthropogenic fertilizers and road salt use are likely causing the change in flux over land use.

Similar to the relationships discussed so far between TDN or ${{\mathrm{NO}}_{3}}^{-}/{{\mathrm{NO}}_{2}}^{-}$ and SC, there also exists a significant relationship between ${{\mathrm{NO}}_{3}}^{-}$ and Cl^−^ fluxes. As shown in Figure 9, datasets collected across varied land use, show that there is a strong relationship between ${{\mathrm{NO}}_{3}}^{-}$ and Cl^−^ even at Pond Branch, a forested reference site for the Baltimore Ecosystem Study (BES) [72]. Fluxes generally increased as land use shifted from rural, to exurban (Baisman Run), with even higher fluxes being recorded at urban catchments (Sligo Creek, Rock Creek, and Accotink Creek). The highest fluxes measured were at the Maumee River, an agricultural catchment in Ohio, where both deicer and fertilizer are used in abundance.

Exurban sites like Baisman Run are especially fascinating as concentrations and fluxes of ${{\mathrm{NO}}_{3}}^{-}$ and Cl^−^ increased from 1998–2018 despite snowfall and road salt use not significantly changing during the period of recording [73]. Concentrations and fluxes were an order of magnitude higher at Baisman Run than in the neighboring Pond Branch, further reinforcing the impact that anthropogenic change has on the biogeochemical mechanisms of affected watersheds [73]. The trends shown in Figure 9 further reinforce that the patterns observed are not only caused by the seasonal build and flush of ${{\mathrm{NO}}_{3}}^{-}$ observed in temperate climates, but also impacts and interactions of other anthropogenic activities [72].

**Figure 10.**
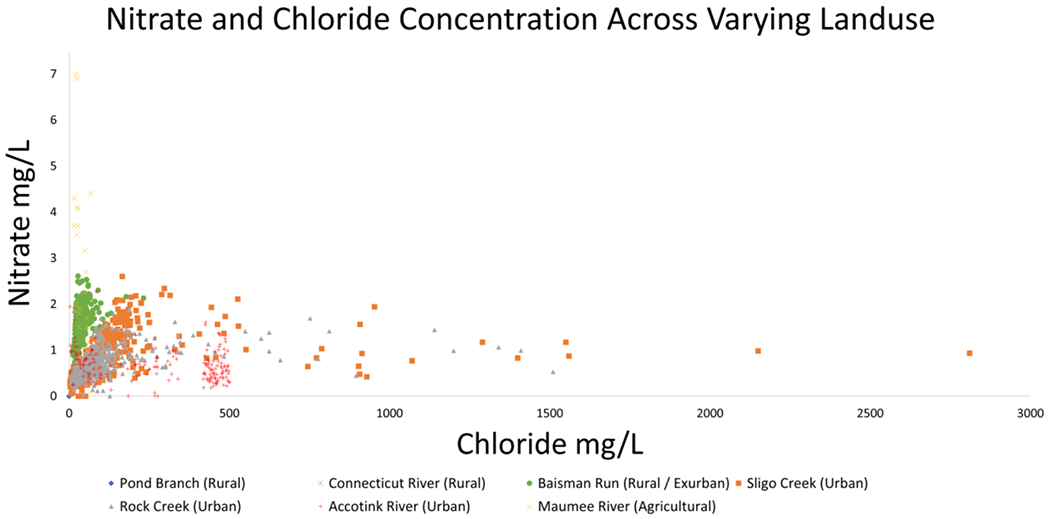
${{\mathrm{NO}}_{3}}^{-}$ and Cl^−^ concentrations show co-mobilization until a plateau. Concentration data from Connecticut River, Sligo Creek, Rock Creek, Accotink Creek, and Maumee River are estimated from concentration and streamflow data from USGS gauge numbers 01161280, 01650800, 01648010, 01654000, and 04183500, respectively. Flux data from Pond Branch and Baisman Run are estimated from concentration data from the Baltimore Ecosystem Study [72] and streamflow data from USGS gauge numbers 01583570 and 01583580, respectively. Concentrations are lowest at rural sites, increasing through urban sites, and reaching a peak at agricultural sites.

${{\mathrm{NO}}_{3}}^{-}$ and Cl^−^ concentrations also illustrate co-mobility in Figure 10. Changes in concentration are not nearly as linear as those observed in Figure 9 with differences in site typology being more pronounced. Maumee River, an agricultural site, had by far the greatest ${{\mathrm{NO}}_{3}}^{-}$ concentrations recorded (up to 7 mg/L) and urban Sligo Creek (with a heavily salted roadway paralleling most of its length) recorded the greatest chloride concentration at nearly 3000 mg/L [72].

## 5. Conclusions

Many monitoring programs mainly study baseflow and targeted rainstorms during fall, summer, and spring, meaning that many important N transport events can be missed during winter snowstorms and episodic road salting. Most of the literature regarding N mobilization focuses on mobilization during rain events but research during the winter months is scarce [74,75]. More work needs to be done in order to capture more detailed snow event data and thus better understand winter N fluxes and cycling in urban watersheds [20,21]. Increased fluxes of N to sensitive, and potentially eutrophic, receiving waters during times of colder temperatures and lower biological uptake could influence downstream transport distances [16]. Increased salinization could also negatively impact the function of any stream restorations present in the affected catchments, as nutrient retention is usually a stated goal of their construction [38,76].

Increased winter monitoring is necessary to better elucidate how road salt inputs may alter the timing and magnitude of TDN mobilization in soils and streams. As there is limited literature on the subject, more data on seasonal trends as well as spatial dynamics of mobilization is needed. High-resolution spatial and temporal data is especially important, as certain trends in mobilization are only exposed by very-high-frequency sampling (≤1 h resolution) (Figure 7). Monitoring for ammonium (${{\mathrm{NH}}_{4}}^{+}$) could also be valuable for future studies as previous research shows that ${{\mathrm{NH}}_{4}}^{+}$ is mobilized by Na^+^ in groundwater [18] and that Na^+^ can alter ${{\mathrm{NH}}_{4}}^{+-}$ cycling as well as mobility in surface waters and soils [48]. Debris dams and other sources of organic matter may act as a sink for ${{\mathrm{NO}}_{3}}^{-}$, but microbial communities residing in them may take time to adapt to the highly saline environments and the associated osmotic stress present in urban streams [27]. In the long term, searching for alternatives to NaCl road salt could help alleviate the situation, but it is unclear if CaCl_2_ or MgCl_2_ are less harmful [13]. The use of brines may be a useful management strategy for the interim as they are already dilute and introduce less salt per unit area [13]. Where available, the use of groundwater shows promise as a possible deicing alternative but its usage appears to be mainly limited to areas of Japan at this time [77]. Managing road salt may also need to be considered in other management strategies for nutrients, metals, and other contaminants due to the potential impacts of road salt on co-mobilization of mixtures of multiple chemicals from soils to streams (e.g., [2–4,9,12,13,36,38]). More experiments, field studies, and snow event monitoring are needed to better elucidate potential interactions between salt and the urban watershed N cycle in a wider geographic range. Data elucidating the proportional N loading from different drivers (fertilizers, legacy sediment, deicers, etc.) would be especially helpful in determining the allocation of remediation efforts.

Effective use of management strategies like brines or alternate deicers requires there to be not only improved monitoring but also ecologically relevant targets for salinity and nutrient loads. For the United States, the EPA suggests a criteria continuous concentration (an estimate of the highest concentration of a pollutant which organisms can be chronically exposed to) for chloride of 230 mg Cl^−^/L and a criteria maximum concentration (an estimate of the highest concentration of a pollutant which organisms can be acutely exposed to) of 860 mg Cl^−^/L [78]. These metrics would be good candidates for re-examination; however, these chloride thresholds are too high to prevent harm to zooplankton communities and in turn damage to the entire food web [78]. The issue of freshwater salinization may be a good candidate for using common pool resource management to generate viable strategies to integrate governance systems, actors, resource units, and resource systems to work towards the common goal of reducing FSS [79].

## Supplementary Material

Supplement1

## Figures and Tables

**Table 1.** USGS monitoring site characteristics are shown below. The state/metro area, latitude, longitude, USGS site number, and monitoring duration are all listed. Rock Creek, Sligo Creek, Scotts Level Branch, Herring Run, and Jones Falls were sampled bi-weekly. Accotink Creek data were obtained from a USGS gauge station. Tidally influenced sites are denoted with a *. All sites were located within the United States of America.

Study Site	State/Metro Area	Latitude	Longitude	USGS Site Number	NLCD 2016 Impervious Surface Cover %	Monitoring Duration
Rock Creek	Washington DC	38°57′36.6″	77°02′31.4″	1648010	18.35	19 October 2017–23 June 2021
Sligo Creek	Washington DC	38°59′10.4″	77°00′17.5″	1650800	27.19	4 October 2017–23 June 2021
Scotts Level Branch	Baltimore MD	39°21′41.7″	76°45′42.3″	1589290	22.24	4 October 2017–15 April 2021
Herring Run	Baltimore MD	39°19′04.7″	76°33′18.5″	1585219	32.76	26 October 2017–3 January 2019
Jones Falls *	Baltimore MD	39°17′02.8″	76°36′13.1″	1589485	21.28	1 November 2017–6 November 2019
Accotink Creek	VA	38°48′46″	77°13′43″	1654000	23.4	5 February 2015–1 Januray 2023

**Table 2.** Snow event sampling conducted with an ISCO automated water sampler. Samples were all collected at Sligo Creek, proximal to USGS station 1650800 in the Washington DC metro area. Δ in specific conductance denotes the minimum and maximum specific conductance values measured during the storm.

Snow Event	Sampling Initiated	Sampling Ceased	N	Δ in Specific Conductance μS/cm
1	1 March 2019, 20:23	2 March 2019, 19:23	24	902–1730
2	16 Decmeber 2020, 10:00	18 Decmeber 2020, 3:10	33	419–5130
3	25 January 2021, 17:30	26 January 2021, 17:00	48	682–3850
4	31 January 2021, 6:00	2 February 2021, 5:00	46	1330–12,500

## Data Availability

Most continuous water quality data used in this paper are on publicly available data sites such as https://waterwatch.usgs.gov/.
