# Supplementary figures and images for: Freshwater Salinization Syndrome Alters Nitrogen Transport in Urban Watersheds
Source: Water (Basel). Author manuscript; Available in PMC 2024 Nov 9. (PMC10831318; doi:10.3390/w15223956)

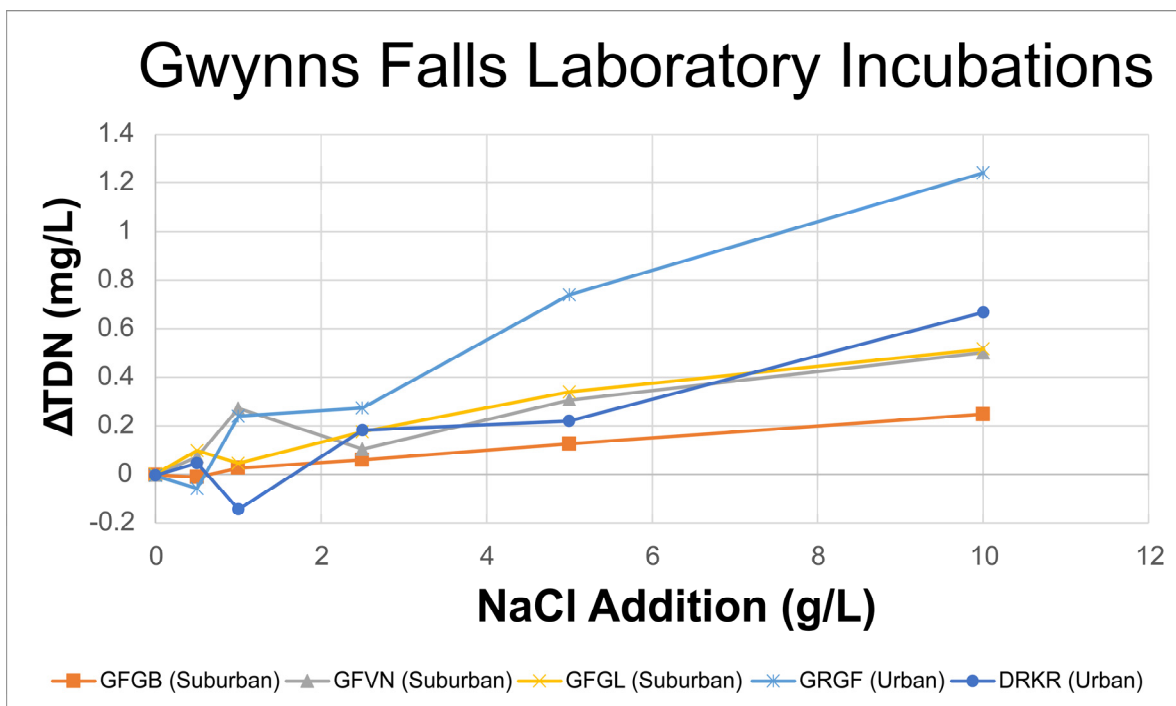

Figure S1. Gwynns Falls Laboratory Incubations.

Supplement: Supplement1 [file NIHMS1945569-supplement-Supplement1.zip › water-2671746-supplementary.pdf]
